# Health Organisation in Great Towns

**Published:** 1920-03-13

**Authors:** 


THE PUBLIC WELL-BEING—(*continued*)

### Health Organisation in Great Towns.

There comes to hand at an opportune time a comprehensive review of the health organisation in some of the great towns. The machine is important in the first instance, but it must be kept in order, not allowed to become obsolete, and the best use made of it. It was, apparently, in this spirit that the Glasgow Corporation, anxious to put its health work on the most efficient and up-to-date basis, appointed a deputation to inquire into the administration of health services to the great towns. The deputation visited London, Birmingham, Sheffield, Manchester, and Liverpool, and returned to Glasgow to recommend the Corporation to reorganise its methods in such a manner as will very materially alter the system of administration. Hitherto the medical officer of health, the sanitary inspector, the veterinary surgeon, and the bacteriologist have all been independent the one of the other, in a way that prevails nowhere else. In making its proposals, the deputation has aimed at concentrating the general direction of all measures necessary for the conservation of the public health. If this were done the various activities of the department would be co-ordinated, overlapping eliminated, and administration generally improved. The proposals, as a whole, are not in operation in any of the cities visited; but, partly in one city and partly in another, have been largely adopted. They mark an appreciable advance in the policy of unification and consolidation. If they are adopted they will lead to more efficient administration, and, at the same time, considerably reduce administrative costs. Some of the appended comparisons are interesting.

### London.

The deputation found that in London the system in operation differs from that prevailing elsewhere, in that public health functions are vested in more than one authority, viz. : The London County Council, the Metropolitan Asylums Board, and the borough councils. In view of this multiplicity of authorities, the proper coordination of public health services involves a problem of great magnitude, and has been reviewed from time to time. At the present time it is again under review, the scheme under consideration being to make the London County Council, which is already the central organising authority for many of the health services in London, the statutory authority to survey the health services within the area, and to supervise and organise those for which the borough councils are responsible. The principle underlying such an arrangement would be that the borough councils should remain responsible for the administration of local services, subject to such measure of coordination and supervision by the Council, as appears necessary and desirable for the different services. The Council would be the sole institutional authority for the area, and it would have power to supervise the health work of the borough councils, and to act in default in the event of neglect. In order to secure the full cooperation of the voluntary hospitals in connection with the scheme, a central body representing the London hospitals has been suggested, with whom the Council might deal with regard to questions affecting the whole of London.

With regard to the present position of the Health Department of the London County Council, the medical officer of health is responsible for the whole of that department, and since 1912 has also been school medical officer. The work of the department is organised in divisions, a medical officer being in charge of each division of the work, responsible to the medical officer of health. Apart from the medical officer of the London County Council, the borough councils have medical officers of health and inspectors of nuisances, the former almost always being responsible for the whole of the work, the latter acting under the general superintendence of the former.

### Birmingham.

In Birmingham the medical officer of health is in charge of the health department, the chief inspector of nuisances acting under his superintendence. Under the medical officer of health are three assistant medical officers, among whom the work of the department is divided up. For a few years before the war the city was divided into four districts, and four assistant medical officers were appointed, who were severally responsible to the medical officer of health for all the work in their respective districts; but this system was departed from during the war, owing to the difficulty at that time of obtaining men with the necessary administrative qualifications. It appears to the deputation to be preferable to have one man responsible for the whole of a district rather than several men for several services in the same district, and recommends that this policy be adopted, as in Glasgow. With regard to the district inspectors, there is no division, as in Glasgow, into epidemic inspectors, nuisance inspectors, etc., each man in his district being responsible for all these duties. This system is much more economical than the system in operation in Glasgow, and no less efficient, and the deputation has no hesitation in recommending its adoption. Apart from the general inspectors, there are special inspectors, who work from the centre, and are not allocated to any particular district of the city. In addition, there is a large female staff in connection with tuberculosis and child welfare, whose duties are specialised. A bacteriologist has just been appointed. He is to be responsible to the medical officer of health. The deputation approves of this arrangement. The expenditure of the department in 1919 represented an assessment of 5.19d. per pound.

### Sheffield.

In Sheffield there are two committees in charge of the ordinary work of the Health Department—viz., the Health Committee and the Hospitals Committee, the latter being independent of the former. The hospitals are not under the medical officer of health. There is a tuberculosis subcommittee of the Health Committee. In addition, a special committee, on which there are co-opted members, manages the King Edward Memorial Hospital for Tuberculous Children. The superintendent of this hospital has also been appointed surgical tuberculosis officer, and as such is independent administratively of the medical officer of health, whereas the tuberculosis officer is responsible to the medical officer of health. The school medical officer is also independent of the medical officer of health, except that he consults with him and acts for him in his absence.

With regard to the inspection of nuisances, instead of one chief inspector being appointed, the city has been divided into five districts, and one inspector has been appointed for each district, with three or four assistants. These inspectors act under the general superintendence of the medical officer of health. The workshops inspectors, the smoke inspectors, and the tuberculosis inspectors have special duties only. Women inspectors are also employed, who have the triple qualification of nurse, midwife, and inspector, so that only duties in connection with child

**THE PUBLIC WELL-BEING—(*continued*).**

welfare, child neglect, tuberculosis, measles, etc. In order to acquire experience of bone tuberculosis they are afforded facilities to go to a hospital for three months, and such nurses as acquire this training are paid an extra £10 of salary. The deputation recommends the appointment of nurses with similar qualifications, instead of nurses for specialised duties.

The veterinary surgeon is on the staff of the medical officer of health, so far as the inspection of milk-cows and meat is concerned, but he has also been appointed veterinary inspector for the purposes of the Diseases of Animals Acts, and as such is independent of the medical officer of health. The expenditure of the department last year represented an assessment of 2.473d. per pound.

MANCHESTER.

The health department in Manchester has recently been organised, and the medical officer of health made head of the department, except that part of the work relating to the inspection of meat. The inspection of milk-cows, however, is under the medical officer of health. The school medical officer is independent of the medical officer of health, and is responsible for the medical inspection of school children, and for infectious diseases in school children, except that their treatment in hospital, the infectious diseases hospitals and the sanatoria comes under the medical officer of health. The medical staff under the medical officer of health consists of two assistant medical officers, who are responsible between them for work other than tuberculosis and child welfare; a tuberculosis officer, who is responsible for tuberculosis work; and a lady medical officer, who is responsible for child welfare work. The chief inspector of nuisances, since the recent reorganisation of the department, acts under the general superintendence of the medical officer of health. The district inspectors' duties are not subdivided, except that there are special food inspectors and smoke inspectors. The expenditure necessitated an assessment of 8.15d. per pound. This did not include expenditure in connection with meat inspection.

LIVERPOOL.

In Liverpool the whole health department is under the medical officer of health, who is also responsible for the medical inspection of school children. The chief inspector of nuisances acts under his superintendence; meat inspection is not carried out by a veterinary surgeon, but by inspectors, who are under the medical officer of health. The veterinary surgeon examines milk-cows when required by the medical officer of health. but he is also appointed veterinary inspector for the purpose of the Diseases of Animals Acts. There is a large staff of male and female inspectors for general sanitary purposes, besides special food inspectors, smoke inspectors, lodging-houses inspectors, shops inspectors, etc. The duties of the visiting nurses in connection with tuberculosis, child welfare, measles, etc., are also specialised, but there is provision for transferring nurses from one branch to another when available. The expenditure represented a rate of 1s. 5d. in the pound. Very full provision has been made for the institutional treatment of tuberculosis in Liverpool.

